# Expected and diagnosed rates of mild cognitive impairment and dementia in the U.S. Medicare population: observational analysis

**DOI:** 10.1186/s13195-023-01272-z

**Published:** 2023-07-22

**Authors:** Soeren Mattke, Hankyung Jun, Emily Chen, Ying Liu, Andrew Becker, Christopher Wallick

**Affiliations:** 1 Center for Improving Chronic Illness Care, USC Dornsife, University of Southern California, 635 Downey Way, #505N, Los Angeles, CA 90089 USA; 2 Department of Health Care Policy, Harvard Medical School, Boston, MA USA; 3 Center for Economic and Social Research, University of Southern California, Los Angeles, CA USA; 4 US Medical Affairs, Genentech, Inc., Roche Group, South San Francisco, CA USA

**Keywords:** Alzheimer’s disease, Dementia, Epidemiology, Health systems research, Medicare, Mild cognitive impairment, Dementia

## Abstract

**Background:**

With the emergence of disease-modifying Alzheimer’s treatments, timely detection of early-stage disease is more important than ever, as the treatment will not be indicated for later stages. Contemporary population-level data for detection rates of mild cognitive impairment (MCI), the stage at which treatment would ideally start, are lacking, and detection rates for dementia are only available for subsets of the Medicare population. We sought to compare documented diagnosis rates of MCI and dementia in the full Medicare population with expected rates based on a predictive model.

**Methods:**

We performed an observational analysis of Medicare beneficiaries aged 65 and older with a near-continuous enrollment over a 3-year observation window or until death using 100% of the Medicare fee-for-service or Medicare Advantage Plans beneficiaries from 2015 to 2019. Actual diagnoses for MCI and dementia were derived from ICD-10 codes documented in those data. We used the 2000–2016 data of the Health and Retirement Study to develop a prediction model for expected diagnoses for the included population. The ratios between actually diagnosed cases of MCI and dementia over number of cases expected, the observed over expected ratio, reflects the detection rate.

**Results:**

Although detection rates for MCI cases increased from 2015 to 2019 (0.062 to 0.079), the results mean that 7.4 of 8 million (92%) expected MCI cases remained undiagnosed. The detection rate for MCI was 0.039 and 0.048 in Black and Hispanic beneficiaries, respectively, compared with 0.098 in non-Hispanic White beneficiaries. Individuals dually eligible for Medicare and Medicaid had lower estimated detection rates than their Medicare-only counterparts for MCI (0.056 vs 0.085). Dementia was diagnosed more frequently than expected (1.086 to 1.104) from 2015 to 2019, mostly in non-Hispanic White beneficiaries (1.367) compared with 0.696 in Black beneficiaries and 0.758 in Hispanic beneficiaries.

**Conclusions:**

These results highlight the need to increase the overall detection rates of MCI and of dementia particularly in socioeconomically disadvantaged groups.

**Supplementary Information:**

The online version contains supplementary material available at 10.1186/s13195-023-01272-z.

## Background

The recent announcement that the amyloid-targeting drugs lecanemab [1] and donanemab [2] met their primary and secondary endpoints in phase 3 trials and the subsequent FDA approval of lecanemab mean that a disease-modifying treatment for Alzheimer’s disease (AD) is available now in the U.S. Both drugs were shown to reduce the speed of cognitive decline in early symptomatic AD, which lends renewed urgency to the timely detection of cognitive decline because these disease-modifying therapies are only indicated in early disease stages (i.e., mild cognitive impairment [MCI] and mild dementia due to AD). However, evidence shows that cognitive decline is commonly diagnosed only once an individual has progressed to an advanced stage of the disease. For example, Thoits et al. [3] found that about 79% of randomly selected patients newly diagnosed at a memory clinic had moderate or severe dementia. These missed and delayed diagnoses have long taken away from patients and families the opportunity to adopt lifestyle changes to reduce the speed of decline [4], start symptomatic medication treatment, and consider measures to increase physical and financial safety and security [5]. But soon, failing to detect early-stage AD will deprive patients of the prospect to alter the course of this devastating illness.

Unfortunately, limited data exist to report the degree of missed diagnoses of MCI, the stage at which AD would ideally be treated [6]. White et al. [7] used data from the Health and Retirement Study (HRS), a nationally representative survey of older US adults that contains cognitive assessments, to estimate that 11.4% of individuals with incident MCI reported receiving a timely diagnosis. Similarly, neuropsychiatric testing data by Savva et al. [8] from the Aging, Demographics, and Memory Study concluded that 15% of participants with a clinical dementia rating of 0.5, a score reflective of MCI, were aware of a diagnosis of cognitive impairment.

More research has been conducted on dementia detection rates. One study linked Medicare claims data to information on 417 patients with a clinical diagnosis of AD in the Consortium to Establish a Registry for Alzheimer’s Disease data and reported that only around 75% of patients had a corresponding diagnosis in claims data in the period from 1991 to 1995 [9], a number similar to the 85% reported by Lee et al. [10] for the 2007 to 2012 period of the same data. Zhu et al. [11] published a dementia prevalence of 12.9% based on cognitive tests and 12.4% based on diagnosis codes in the 20% sample of Medicare fee-for-service (FFS) beneficiaries in 2012. Jutkowitz et al. [12] found considerably lower dementia diagnosis rates of 5.6% and 6.5% in 2014 and 2016, respectively, in a convenience sample of 3 Medicare Advantage Plans.

However, those studies commonly use older data and/or are limited to subsets of either the Medicare FFS population or members of Medicare Advantage Plans, which is important as the decision to enroll in a Medicare Advantage Plan is not random [13, 14]. More critically, most prior studies are confined to identifying the prevalence of dementia diagnoses, and—to our knowledge—no study has looked into the gap at the stage of MCI in the full Medicare population.

Thus, the objective of this study is to derive contemporary population-level diagnosis rates of MCI and dementia documented in the full Medicare population and compare these with the expected rates using population survey data from individuals who had undergone cognitive assessment. We are using the 100% sample from 2015 to 2019 for both Medicare FFS and Medicare Advantage Plans to achieve full population coverage to determine observed diagnosis rates. We are using data from the 2000 to 2016 waves of the HRS to derive the prediction model for expected diagnosis rates.

## Methods

### Medicare data

The analyses include the 100% sample for beneficiaries aged 65 and older enrolled in Medicare FFS or a Medicare Advantage Plan, who were nearly continuously enrolled either for at least 3 years or until death. Following the coverage definition used by the Chronic Conditions Data Warehouse (CCW), our definition of *nearly continuous enrollment* requires an average of 11 months of both parts A and B or part C coverage each year (at least 33 out of a possible 36 months) or, if the beneficiary died during the third year of the surveillance period, with fully continuous parts A and B or part C coverage and no interruption until the month of death. Across all 3-year windows from 2015 to 2019, this restriction excluded 13.2% (*n* = 6,890,000) of beneficiaries aged 65 and older during each of the 3 years.

Advantage Plan enrollment was defined as having at least 2 months of enrollment [15], as switching outside of the open enrollment period is uncommon [16, 17]. We analyzed claims and encounter data for inpatient and outpatient facilities, carriers, and skilled nursing facilities and the corresponding enrollment data for 2015 to 2019. Data were accessed through the Centers for Medicare & Medicaid Services Virtual Research Data Center, and the study protocol and data protection procedures were approved by our Institutional Review Board (UP-21-00441) under expedited review and with a waiver for informed consent and HIPAA (Health Insurance Portability and Accountability Act) authorization. Data were processed and analyzed with SAS 7.15 (SAS Institute Inc.), and all statistical analyses were conducted with Stata 16 (StataCorp LLC).

### Calculation of diagnosed prevalence in claims data

To identify persons diagnosed with dementia, we modified the algorithm published by the CCW [18] based on a recent study by Festa et al., [19] which found that noncognitive and unspecific codes in the CCW algorithm, as well as diagnoses made during a single inpatient or skilled nursing facility episode, were likely to be false positives. The list of included and removed codes is shown in Additional file 1: Table S1 and Table S2. We required 2 claims with the remaining codes on separate days in either setting over rolling windows of 3 years. We also used the original CCW algorithm for comparison. No definitions for MCI are currently published by the CCW, so we identified the diagnosis based on ICD-10-CM code G31.84 (i.e., mild cognitive impairment of uncertain or unknown etiology) and the ICD-9-CM code 331.83 (i.e., mild cognitive impairment), also requiring 2 claims on separate days. The rates were calculated for each 3-year rolling window for which we had complete data—that is, 2015 to 2017, 2016 to 2018, and 2017 to 2019.

As some persons may meet the criteria for both MCI and dementia, we applied the following assignment rules to avoid double counting: If a person was uniquely assigned to either MCI or dementia during the midpoint year, we used that assignment. If not, we based the assignment on the latest claim with a diagnosis of MCI or dementia in the midpoint year. If neither diagnosis was documented during the midpoint year, we based the assignment on the claim closest to the midpoint year or, for ties, in the earlier year. Among this group, accounting for about 10% of those who had either diagnosis, around 40% were assigned to MCI and 60% to dementia.

### Development and validation of the model to generate expected rates

To estimate the underlying prevalence of MCI and dementia based on cognitive assessments, we used HRS data from 2000 to 2016 [11]. We applied the Langa-Kabeto-Weir Classification of Cognitive Function [20] to categorize participants as cognitively normal, as having cognitive impairment but no dementia ([CIND] with CIND representing MCI), or as having dementia. These classifications were based on cognitive assessments of self-respondents and proxy interviews, usually with a spouse or other family member, if a respondent was unable or unwilling to do an interview.

We used probit models to separately predict CIND (vs cognitively normal) and dementia (vs cognitively normal) using variables that are present both in the HRS and Medicare data to allow using the model to generate expected rates in the Medicare population. Predictors included sex, age groups (50–54, 55–59, 60–64, 65–69, 70–74, 75–79, 80–84, and ≥ 85 years), race and ethnicity (non-Hispanic [NH] White, Black, Hispanic, and others), dual eligibility status (individuals covered by both Medicare and Medicaid), and a linear trend for year to account for the secular decline in dementia incidence [21]. Our analytic sample consisted of HRS participants aged 65 and older.

Model calibration was conducted using the 2000 to 2014 data. To validate the calibration, we applied the derived regression weights to the 2016 HRS participants, calculated their probabilities of having CIND or dementia, and used receiver operating characteristic curves to quantify the prediction accuracy of the model. The derivation of national estimates of the rate of Medicare beneficiaries with CIND and dementia based on these predicted probabilities is detailed in Additional file 1: Methods. Sampling weights were used in the analysis.

As the person-level predictors in the HRS-based models are also available in Medicare enrollment data, the estimated regression weights can be applied to Medicare data to generate expected diagnosis rates for MCI and dementia, accounting for potentially different demographic compositions and a secular trend if additional years of data are used. The ratio between the observed rates based on diagnoses documented in the claims data and the expected rates predicted by the model provides a measure for potential gaps in diagnoses. Such observed to expected (O/E) ratios are frequently used in quality measurement [22]. The O/E ratio can be interpreted as the proportion of expected cases that were diagnosed or the detection rate.

### Role of the funding source

This research was funded by a contract from Genentech, a member of the Roche Group, to the University of Southern California. The sponsor provided comments on a draft of the paper, but the authors had full control of the design, analysis, final manuscript, and decision to submit.

## Results

### Diagnosed rates in Medicare data

Table 1 shows the observed rates of MCI and dementia diagnosis in the Medicare population. Rates for MCI increased by about 10% for each observation window or 0.3 percentage points from 0.012 to 0.015, whereas dementia rates declined slightly from 0.098 to 0.095. From 2017 to 2019, rates for both MCI and dementia increased with age (Table 2). Dementia (0.108 vs 0.079), but not MCI (0.016 vs 0.015), was more frequently diagnosed in women than in men (Table 3). Dementia, but not MCI, was also more frequently diagnosed in Black and Hispanic individuals than in NH White individuals (Table 4). Dually eligible beneficiaries had substantially higher diagnosis rates for dementia compared with their Medicare-only counterparts (0.213 vs 0.079), but similar rates for MCI (0.017 vs 0.015) and diagnosis rates for both stages of cognitive impairment were similar for individuals enrolled in Medicare FFS and Medicare Advantage Plans (Table 5).

**Table 1:** Observed and expected rates and detection rates for MCI and dementia over time

	**Observation window**		
	**2015–2017**	**2016–2018**	**2017–2019**
**Included beneficiaries**	38,739,387	39,965,446	41,205,474
**MCI**			
Observed rate	0.012	0.014	0.015
Expected rate	0.199	0.197	0.196
Detection rate^a^	0.062	0.071	0.079
**Dementia**			
Observed rate	0.098	0.097	0.095
Expected rate	0.090	0.088	0.086
Detection rate^a^	1.086	1.093	1.104

*MCI* Mild cognitive impairment

^a^The detection rate is the ratio of the observed to the expected rate, where the expected rate is computed based on a predictive model

**Table 2:** Observed and expected rates and detection rates for MCI and dementia by age, 2017 to 2019

	**Age (in years)**				
	**65–69**	**70–74**	**75–79**	**80–84**	**≥ 85**
**Included beneficiaries**	8,913,079	12,030,038	8,682,044	5,750,802	5,829,511
**MCI**					
Observed rate	0.008	0.011	0.017	0.023	0.026
Expected rate	0.127	0.155	0.202	0.256	0.315
Detection rate^a^	0.060	0.071	0.086	0.090	0.083
**Dementia**					
Observed rate	0.024	0.039	0.079	0.149	0.294
Expected rate	0.028	0.041	0.072	0.122	0.256
Detection rate^a^	0.831	0.942	1.110	1.221	1.147

*MCI* Mild cognitive impairment

^a^The detection rate is the ratio of the observed to the expected rate, where the expected rate is computed based on a predictive model

**Table 3:** Observed and expected rates and detection rates for MCI and dementia by sex, 2017 to 2019

	**Sex**	
	**Female**	**Male**
**Included beneficiaries**	17,796,969	23,408,505
**MCI**		
Observed rate	0.016	0.015
Expected rate	0.189	0.204
Detection rate^a^	0.084	0.074
**Dementia**		
Observed rate	0.108	0.079
Expected rate	0.095	0.075
Detection rate^a^	1.141	1.043

*MCI* Mild cognitive impairment

^a^The detection rate is the ratio of the observed to the expected rate, where the expected rate is computed based on a predictive model

**Table 4:** Observed and expected rates and detection rates for MCI and dementia by race and ethnicity, 2017 to 2019

	**Race and ethnicity**			
	**NH White**	**Black**	**Hispanic**	**Others**^**a**^
**Included beneficiaries**	31,701,890	3,553,686	3,489,177	2,460,721
**MCI**				
Observed rate	0.016	0.013	0.016	0.011
Expected rate	0.163	0.332	0.338	0.218
Detection rate^b^	0.098	0.039	0.048	0.050
**Dementia**				
Observed rate	0.093	0.122	0.112	0.071
Expected rate	0.068	0.175	0.148	0.112
Detection rate^b^	1.367	0.696	0.758	0.630

*NH* Non-Hispanic, *MCI* Mild cognitive impairment

^a^Includes unknown race/ethnicity

^b^The detection rate is the ratio of the observed to the expected rate, where the expected rate is computed based on a predictive model

**Table 5:** Observed and expected rates and detection rates for MCI and dementia by dual eligibility status and coverage type, 2017 to 2019

	**Dually eligible**^**a**^	**Medicare only**	**Fee-for-service**	**Medicare Advantage**
**Included beneficiaries**	5,102,819	36,102,655	22,957,446	18,248,028
**MCI**				
Observed rate	0.017	0.015	0.016	0.015
Expected rate	0.308	0.180	0.186	0.207
Detection rate^b^	0.056	0.085	0.084	0.073
**Dementia**				
Observed rate	0.213	0.079	0.096	0.095
Expected rate	0.250	0.063	0.082	0.092
Detection rate^b^	0.855	1.243	1.167	1.034

*MCI* Mild cognitive impairment

^a^Dually eligible for Medicare and Medicaid

^b^The detection rate is the ratio of the observed to the expected rate, where the expected rate is computed based on a predictive model

### Estimated rates based on HRS data

The estimated regression weights derived from the 2000 to 2014 HRS data are listed in Additional file 1: Table S3. Applying these estimates to 2016 HRS data showed that the predicted rate of MCI in 2016 was 0.176 compared with an observed rate of 0.175; for dementia, the predicted and observed rates were 0.082 and 0.078, respectively. The area under the receiver operating characteristic curve was 0.670 for separating MCI from being cognitively normal and 0.772 for separating dementia from being cognitively normal; accuracy rates show that 68.6% and 79.2% of the sample were correctly classified for MCI and dementia, respectively (Additional file 1: Table S4 and Fig. 1).

### Expected diagnosis rates and detection rates in Medicare data

The expected prevalence shows a slight decline for both MCI (0.199 to 0.196) and dementia (0.090 to 0.086) from 2015 to 2019 (Table 1). The detection rates for both stages increased during the same period, albeit faster for MCI than dementia with average increases of 20% (1.7 percentage points) and 0.85% (1.8 percentage points), respectively. Put differently, of the estimated 8 million Medicare beneficiaries with MCI, 7.4 million were undiagnosed, while diagnosis rates for dementia were about 10% too high. Table 2 documents that detection rates increase with age, except for the oldest cohort, and Table 3 shows that these ratios are higher for women than for men. Detection rates for MCI in NH White Medicare beneficiaries were 2- to 3-fold those of Black and Hispanic beneficiaries and those with other or unknown race or ethnicity, and detection rates for dementia in NH White Medicare beneficiaries were 2.5-fold those of Black and Hispanic beneficiaries (Table 4). In other words, only around two-thirds to three-quarters of the expected non-White individuals with dementia were diagnosed, whereas diagnosis rates for NH White individuals were higher than predicted. Dually eligible individuals had lower estimated detection rates than their Medicare-only counterparts for MCI (detection rate 0.056 vs 0.085) and dementia (detection rate 0.855 vs 1.243). Beneficiaries in Medicare FFS were more likely to have their cognitive impairment detected than those in Medicare Advantage Plans, with a detection rate of 0.084 vs 0.073 for MCI and 1.167 vs 1.034 for dementia (Table 5).

## Discussion

We determined the observed rates of MCI and dementia diagnosis in the full US Medicare population aged 65 and older and compared those with expected rates based on a predictive model. Only 7.9% of expected MCI cases have been diagnosed in the most recently available data covering 2017 to 2019. This estimate implies that approximately 7.4 million Medicare beneficiaries 65 and older live with undiagnosed MCI today. If the same rate of underdiagnosis were applied to a younger cohort, there would be another 3 million undiagnosed Americans between the ages 50 and 64 [23]. In other words, there may be around 10 million Americans with undiagnosed MCI, and around half of them would have the AD pathology [24]. While it is difficult to estimate which proportion of those would benefit from a disease-modifying treatment for AD, the magnitude of the gap is concerning. Our estimate is in line with those of other studies. Borson et al. [25] reported that primary care physicians correctly identified just 6% of MCI cases in a small sample of 371 patients. A similar study in Germany found that only 11 to 12% of MCI cases were detected by primary care physicians [26]. The aforementioned studies by White et al. [7] and Savva et al. [8] estimated that the rates of self-reported MCI diagnosis were 11.4% and 15%, respectively.

Thus, increased efforts to detect MCI earlier are dearly needed, especially for socioeconomically disadvantaged groups, which have a higher risk of missed diagnosis—and a recently published consensus recommendation proposed several steps to achieve this [27]. The Medicare Annual Wellness Visit, which requires a cognitive assessment, might increase detection rates, but uptake remains limited, and a recent study reported that cognitive state was formally assessed in less than one-third of visits [28].

In contrast, dementia appears to be overdiagnosed in Medicare beneficiaries by about 10%, even when we used a more stringent identification algorithm modified following the publication by Festa et al. [19]. Based on the original CCW algorithm, the observed rate would have been 0.106 and the detection rate 1.23, suggesting a 23% rate of overdiagnosis. This dementia detection rate is much higher than the 38% reported by a meta-analysis of US-based studies prior to 2007 [29] and the July 2022 estimate of 62% from the National Health Service in England [30], but close to a 2.4% overdiagnosis rate estimated by Zhu et al., [11] which used 2012 HRS data linked to Medicare claims.

However, socioeconomically disadvantaged populations have much lower dementia detection rates in spite of their greater disease burden [31] and higher rates of risk factors [32], which mirrors the results published by others, who compared HRS data to diagnoses in Medicare claims. Gianattasio et al. [33] had estimated that Black individuals had nearly twice the risk of underdiagnosis as NH White individuals between 2000 and 2010. Zhu et al. [11] found that the discrepancy shrank but did not disappear fully by 2014, as did Lin et al. [34]. Although our diagnosis rates were substantially higher than expected rates in NH White individuals, they were only around two-thirds to three-quarters of expected rates in other ethnic and racial groups for the 2017 to 2019 window. Similarly, dually eligible beneficiaries, one of the most vulnerable groups, have diagnosis rates of 85.5% vs the expected rates. It is somewhat unexpected that members of Medicare Advantage Plans have lower detection rates than those in Medicare FFS, because Medicare Advantage Plans commonly include clinical home visits that lead to the detection of undiagnosed disease [35], and a dementia diagnosis is part of the risk adjustment scheme that sets capitation rates for plans. However, the finding may be reflective of the selective switching of Medicare patients with dementia from Advantage Plans to FFS [36].

### Limitations

These results should be interpreted within the context of the study limitations. We acknowledge that a predictive model based on demographic information alone has only adequate accuracy and it could be improved by incorporating clinical characteristics of the individuals. Also, we estimated the expected prevalence of MCI based on cognitive test scores, which is not the same as a true clinical diagnosis. However, our predicted number of 8.06 million cases is close to the 7.95 million predicted based on a widely recognized meta-analysis by Petersen et al., [37] suggesting our estimated detection rate should be reasonably accurate. In contrast, it is possible that our model underpredicts dementia prevalence, because our expected rate of 0.086 is slightly lower than the rates reported for the US population aged 65 and older by Rajan et al. [38] (0.113) and Manly et al. [39] (0.100), which are closer to the observed rate of 0.096. Our classification algorithm for the cognitive state using the HRS data may overclassify dementia in minority populations [33], which could explain part of their lower detection rates. Similarly, the classification algorithm based on claims data may have misclassified some individuals [40]. We did not account for switching between FFS and Medicare Advantage within an observation window, as switching rates tend to be low, between 1 and 6% [16, 17]. Diagnoses may have been communicated but not documented in claims data because of concerns for stigma and loss of driver’s license, but lack of documentation still represents a problem, as it may limit the clinician’s ability to prescribe symptomatic medications and connect patients with support services. We would also expect the reluctance to document to be greater for dementia than for MCI, which is the opposite of what the data show.

## Conclusions

Overall, our findings represent—to our knowledge—the first assessment of diagnosis rates of MCI and dementia relative to estimated prevalence for the full Medicare population. These results point to a need to improve early detection of cognitive impairment, particularly in socioeconomically disadvantaged groups.

## Supplementary Information

**Additional file 1:** **Table S1.** Modification of Chronic Conditions Data Warehouse ICD-10 codes for dementia diagnosis in Medicare data. **Table S2.** ICD-9 codes for dementia diagnosis in Medicare data. **Methods. **Derivation of expected probabilities of having MCI, dementia, or normal cognitive state from probit model predictions. **Table S3.** Probit model estimates using 2000 to 2014 HRS data from respondents aged 65 and older. **Table S4.** Validation of predicted rates against observed rates using 2016 HRS data among respondents aged 65 and older (*N*=9,808). **Fig. S1.** Area under the receiver operating characteristic curve when the predicted probabilities of having MCI or dementia (vs being cognitively normal) are compared to the cognitive states determined by cognitive assessments and information reports, using 2016 HRS data from respondents aged 65 or older.

## Data Availability

The raw Medicare data and person-level analytic files that support the findings of this study may not be shared because of privacy protection. Selected summary data beyond those included in the manuscript are available from the corresponding author, SM, upon reasonable request.

## Abbreviations

AD: Alzheimer’s disease
CCW: Chronic Conditions Data Warehouse
CIND: Cognitive impairment but no dementia
FFS: Fee-for-service
HIPAA: Health Insurance Portability and Accountability Act
HRS: Health and Retirement Study
MCI: Mild cognitive impairment
NH: Non-Hispanic
O/E: Observed over expected
