# Supplementary material for: Expected and diagnosed rates of mild cognitive impairment and dementia in the U.S. Medicare population: observational analysis
Source: Alzheimers Res Ther. 2023 Jul 22;15:128. doi: 10.1186/s13195-023-01272-z (PMC10362635; doi:10.1186/s13195-023-01272-z)
Supplement: Supplementary file 1 — Additional file 1: Table S1. Modification of Chronic Conditions Data Warehouse ICD-10 codes for dementia diagnosis in Medicare data. Table S2. ICD-9 codes for dementia diagnosis in Medicare data. Methods. Derivation of expected probabilities of having MCI, dementia, or normal cognitive state from probit model predictions. Table S3. Probit model estimates using 2000 to 2014 HRS data from respondents aged 65 and older. Table S4. Validation of predicted rates against observed rates using 2016 HRS data among respondents aged 65 and older (N=9,808). Fig. S1. Area under the receiver operating characteristic curve when the predicted probabilities of having MCI or dementia (vs being cognitively normal) are compared to the cognitive states determined by cognitive assessments and information reports, using 2016 HRS data from respondents aged 65 or older. [file 13195_2023_1272_MOESM1_ESM.docx]

# **Supplement**

# **eTable 1.** Modification of Chronic Conditions Data Warehouse ICD-10 codes for dementia diagnosis in Medicare data

# **eTable 2.** ICD-9 codes for dementia diagnosis in Medicare data

# **eMethods.** Derivation of expected probabilities of having MCI, dementia, or normal cognitive state from probit model predictions

# **eTable 3.** Probit model estimates using 2000 to 2014 HRS data from respondents aged 65 and older

# **eTable 4.** Validation of predicted rates against observed rates using 2016 HRS data among respondents aged 65 and older (N=9,808)

# **eFigure 1.** Area under the receiver operating characteristic curve when the predicted probabilities of having MCI or dementia (vs being cognitively normal) are compared to the cognitive states determined by cognitive assessments and information reports, using 2016 HRS data from respondents aged 65 or older

## **eTable 1. Modification of Chronic Conditions Data Warehouse ICD-10 codes for dementia diagnosis in Medicare data**

Festa et al^15^ found that noncognitive and unspecific codes in the CCW algorithm for ICD-10, as well as diagnoses made during a single inpatient or SNF episode were more likely to be false-positives. We thus removed the diagnosis codes that are greyed out in the below list and required 2 claims with any of the remaining codes on separate days in either setting.

| **ICD-10 code** | **Description** |
| --- | --- |
| F01.50 | Vascular dementia, unspecified severity, without behavioral disturbance, psychotic disturbance, mood disturbance, and anxiety |
| F01.51 | Vascular dementia, unspecified severity, with behavioral disturbance |
| F02.80 | Dementia in other diseases classified elsewhere, unspecified severity, without behavioral disturbance, psychotic disturbance, mood disturbance, and anxiety |
| F02.81 | Dementia in other diseases classified elsewhere, unspecified severity, with behavioral disturbance |
| F03.90 | Unspecified dementia, unspecified severity, without behavioral disturbance, psychotic disturbance, mood disturbance, and anxiety |
| F03.91 | Unspecified dementia, unspecified severity, with behavioral disturbance |
| F04 | Amnestic disorder due to known physiological condition |
| F05 | Delirium due to known physiological condition |
| F06.1 | Catatonic disorder due to known physiological condition |
| F06.8 | Other specified mental disorders due to known physiological condition |
| G13.8 | Systemic atrophy primarily affecting central nervous system in other diseases classified elsewhere |
| G30.0 | Alzheimer's disease with early onset |
| G30.1 | Alzheimer's disease with late onset |
| G30.8 | Other Alzheimer's disease |
| G30.9 | Alzheimer's disease, unspecified |
| G31.1 | Senile degeneration of brain, not elsewhere classified |
| G31.2 | Degeneration of nervous system due to alcohol |
| G31.01 | Pick's disease |
| G31.09 | Other frontotemporal neurocognitive disorder |
| G94 | Other disorders of brain in diseases classified elsewhere |
| R41.81 | Age-related cognitive decline |
| R54 | Age-related physical debility |

Abbreviations: CCW, Chronic Conditions Data Warehouse; SNF, skilled nursing facility.

## **eTable 2. ICD-9 codes for dementia diagnosis in Medicare data**

For ICD-9, we used the codes based on a publication by Albrecht et al (doi: [10.1097/WAD.0000000000000295](https://doi.org/10.1097%2FWAD.0000000000000295)), except for codes that indicate senile psychosis (290.80 and 290.90).

| **ICD-9 code** | **Description** |
| --- | --- |
| 331.0 | Alzheimer’s disease |
| 331.82 | Lewy-body associated dementia |
| 331.10, 331.11, 331.19 | Frontotemporal dementia |
| 290.40, 290.41, 290.42, 290.43 | Vascular dementia |
| 290.0x, 290.10, 290.11, 290.12, 290.13, 290.20, 290.21, 290.3x, 290.80 , 290.90, 294.0x, 294.10, 294.11, 294.20, 294.21, 294.8, 331.2, 331.7, 797.xx | Non-specific dementias |

## **eMethods. Derivation of expected probabilities of having MCI, dementia, or normal cognitive state from probit model predictions**

In the probit model calibration, we separately predicted having MCI vs being cognitively normal (CN) and having dementia vs CN. In other words, the predicted values from each equation reflect the expected proportion of MCI among MCI and CN subsamples and expected proportion of dementia among dementia and CN subsamples. To obtain the expected rates of the 3 cognitive states over the entire sample, additional conversion is needed, and we show the mathematical derivation below.

Let $p^{cn}$, $p^{mci}$, and $p^{dem}$ represent the expected proportions of CN, MCI, and dementia, respectively, over the entire sample, and $e^{mci}$ and $e^{dem}$ denote the predicted probabilities from the probit models. We then have

$e^{mci}=$ $p^{mci}/(p^{mci}+p^{cn})$ --- (1)

$e^{dem}=$ $p^{dem}/(p^{dem}+p^{cn})$. --- (2)

With simple transformation, these can be rewritten as:

$p^{mci}=p^{cn}\cdot e^{mci}/\left( 1-e^{mci} \right)$ --- (3)

$p^{dem}=p^{cn}\cdot e^{dem}/\left( 1-e^{dem} \right)$. --- (4)

Given that

$p^{cn}+p^{mci}+p^{dem}=1$, --- (5)

plugging (3) and (4) into (5), and solving the equation will result in:

$p^{cn}=\frac{\left( 1-e^{mci} \right)\left( 1-e^{dem} \right)}{e^{dem}\left( 1-e^{mci} \right)+e^{mci}\left( 1-e^{dem} \right)+\left( 1-e^{mci} \right)\left( 1-e^{dem} \right)}$ --- (6)

Further plugging the computed $p^{cn}$ into (3) and (4) will obtain $p^{mci}$ and $p^{dem}$, respectively.

Abbreviations: MCI, mild cognitive impairment.

## **eTable 3. Probit model estimates using 2000 to 2014 HRS data from respondents aged 65 and older**

|  | **MCI^a^** | | **Dementia^a^** | |
| --- | --- | --- | --- | --- |
|  | *b* | 95% CI | *b* | 95% CI |
| Constant | 18.5195 | (13.354-23.685) | 25.0945 | (17.648-32.541) |
| Sex: male^b^ | 0.1141 | (0.084-0.144) | 0.0340 | (-0.01-0.079) |
| Age groups^c^ |  |  |  |  |
| 70-74 | 0.1890 | (0.16-0.218) | 0.2882 | (0.242-0.335) |
| 75-79 | 0.4261 | (0.392-0.46) | 0.6654 | (0.614-0.717) |
| 80-84 | 0.6798 | (0.641-0.719) | 1.0610 | (1.004-1.118) |
| ≥85 | 1.0763 | (1.032-1.12) | 1.7593 | (1.699-1.819) |
| Race & ethnicity^d^ |  |  |  |  |
| Non-Hispanic Black | 0.7324 | (0.688-0.777) | 0.8200 | (0.758-0.882) |
| Hispanic | 0.6548 | (0.599-0.711) | 0.5745 | (0.494-0.655) |
| Other | 0.2826 | (0.176-0.389) | 0.3345 | (0.181-0.488) |
| Dually eligible^e^ | 0.5247 | (0.477-0.573) | 0.9904 | (0.933-1.047) |
| Year | -0.0099 | (-0.012--0.007) | -0.0136 | (-0.017--0.01) |
| Pseudo R-squared | 0.0988 |  | 0.2545 |  |
| N^f^ | 77,210 |  | 68,616 |  |

^a^Compared to being cognitively normal. The standard errors are clustered at the household level.

^b^Compared to female.

^c^Compared to respondents aged 65 to 69 years.

^d^Compared to non-Hispanic White.

^e^Compared to Medicare-only beneficiaries.

^f^Sample size reflects cumulative numbers of observations across 2000 to 2014.

Abbreviations: HRS, Health and Retirement Study; MCI, mild cognitive impairment.

## **eTable 4. Validation of predicted rates against observed rates using 2016 HRS data among respondents aged 65 and older (N=9,808)**

|  | **MCI** | | **Dementia** | |
| --- | --- | --- | --- | --- |
|  | Rate | 95% CI | Rate | 95% CI |
| Observed^a^ | 0.1753 | (0.167-0.184) | 0.0781 | (0.072-0.084) |
| Predicted^b^ | 0.1764 | (0.174-0.179) | 0.0816 | (0.079-0.084) |
| AUC^c^ | 0.6702 |  | 0.7715 |  |
| Sensitivity^d^ | 49.26% |  | 54.69% |  |
| Specificity^d^ | 73.68% |  | 81.93% |  |
| PPV^d^ | 32.76% |  | 25.15% |  |
| NPV^d^ | 84.80% |  | 94.22% |  |
| Accuracy^d^ | 68.64% |  | 79.21% |  |

^a^Computed based on the weighted proportion of 2016 HRS respondents who were classified to having MCI or dementia based on their cognitive assessments or informant reports.

^b^Computed based on the weighted average of the respondents’ predicted probabilities of having MCI or dementia by applying the estimates from the probit calibration (using 2000-2014 data) to respondents in 2016 and converting the predicted values as shown in Appendix A.

^c^Area under the receiver operating curve when the predicted probabilities of having MCI or dementia (vs being cognitively normal) are compared against the cognitive states determined by cognitive assessments or informant reports.

^d^Sensitivity, specificity, PPV, NPV, and accuracy is based on a cutoff of 0.3.

Abbreviations: AUC, area under the curve; MCI, mild cognitive impairment; NPV, negative predictive value; PPV, positive predictive value.

## **eFigure 1. Area under the receiver operating curve when the predicted probabilities of having MCI or dementia (vs being cognitively normal) are compared to the cognitive states determined by cognitive assessments and information reports, using 2016 HRS data from respondents aged 65 or older**

## MCI vs being cognitively normal

## Dementia vs being cognitively normal

Abbreviations: HRS, Health and Retirement Study; MCI, mild cognitive impairment; ROC, receiver operating characteristic.
